# Towards unravelling *Wolbachia* global exchange: a contribution from the *Bicyclus* and *Mylothris* butterflies in the Afrotropics

**DOI:** 10.1186/s12866-020-02011-2

**Published:** 2020-10-20

**Authors:** Anne Duplouy, Robin Pranter, Haydon Warren-Gash, Robert Tropek, Niklas Wahlberg

**Affiliations:** 1 Department of Biology, Lund University, Lund, Sweden; 2 Organismal and Evolutionary Biology Research Programme, The University of Helsinki, Helsinki, Finland; 3 Overstrand Mansions, Prince of Wales Drive, London, SW11 4EU UK; 4 Department of Ecology, Faculty of Science, Charles University, Prague, Czech Republic; 5 Institute of Entomology, Biology Centre of the Czech Academy of Sciences, Ceske Budejovice, Czech Republic

**Keywords:** Symbiosis, Vertical transmission, Horizontal transfer, Phylogeny, Lepidoptera, Interspecific interactions

## Abstract

**Background:**

Phylogenetically closely related strains of maternally inherited endosymbiotic bacteria are often found in phylogenetically divergent, and geographically distant insect host species. The interspecies transfer of the symbiont *Wolbachia* has been thought to have occurred repeatedly, facilitating its observed global pandemic. Few ecological interactions have been proposed as potential routes for the horizontal transfer of *Wolbachia* within natural insect communities. These routes are however likely to act only at the local scale, but how they may support the global distribution of some *Wolbachia* strains remains unclear.

**Results:**

Here, we characterize the *Wolbachia* diversity in butterflies from the tropical forest regions of central Africa to discuss transfer at both local and global scales. We show that numerous species from both the *Mylothris* (family Pieridae) and *Bicyclus* (family Nymphalidae) butterfly genera are infected with similar *Wolbachia* strains, despite only minor interclade contacts across the life cycles of the species within their partially overlapping ecological niches. The phylogenetic distance and differences in resource use between these genera rule out the role of ancestry, hybridization, and shared host-plants in the interspecies transfer of the symbiont. Furthermore, we could not identify any shared ecological factors to explain the presence of the strains in other arthropod species from other habitats, or even ecoregions.

**Conclusion:**

Only the systematic surveys of the *Wolbachia* strains from entire species communities may offer the material currently lacking for understanding how *Wolbachia* may transfer between highly different and unrelated hosts, as well as across environmental scales.

**Supplementary information:**

**Supplementary information** accompanies this paper at 10.1186/s12866-020-02011-2.

## Background

**Fig. 1:**
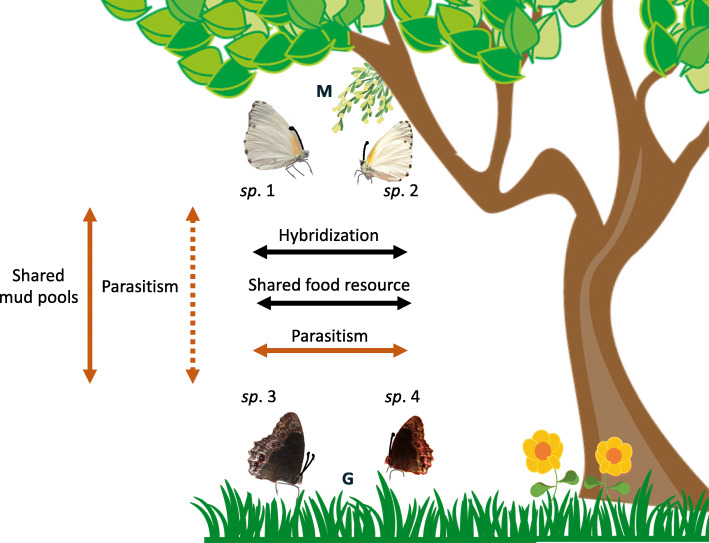
The micro-habitat of the *Mylothris* and *Bicyclus* butterflies in an African tropical forest habitat, and the diverse potential routes of transfer of *Wolbachia* between and within the butterfly species. In orange the routes that remain to be fully tested, with unlikely routes in dashed lines. (M): Mistletoe, the host-plant of *Mylothris* butterflies, and (G): Grass, the host-plant of *Bicyclus* butterflies. Butterfly images modified from pictures by authors HWG and RT

The maternally inherited endosymbiont *Wolbachia* is present in more than 20% of all insect species, making this bacterium one of the most successful organisms on Earth [1–3]. Although host-*Wolbachia* co-divergence is relatively common between Nematode hosts and their *Wolbachia* strains, similar examples of co-divergence between insect hosts and their *Wolbachia* strains remain scarce ([4, 5], but see [6]). These patterns thus suggest that *Wolbachia* may have jumped horizontally between host species throughout the ~ 400 million years of the symbiont evolutionary history [5, 7–10]. Hybridization events, followed by introgression between closely related species have been shown to support the interspecies transfer of various genetic entities, including *Wolbachia* [11–13]. Although recent common ancestry is an obvious reason to why two species can carry the same symbionts, studies have shown that it is not the only one (Fig. 1).

Various ecological interactions between hosts appear to support the horizontal transfer (HT) of *Wolbachia* between highly divergent species. Through the study of Diptera associated with fleshy mushrooms, Stahlhut et al. [14] suggested that the HT through species hybridization occurs between species of this community, but that shared food-resources may also provide an efficient support for the horizontal movement of the bacterium between divergent host species. Similar conclusions were drawn from the study of an insect community feeding on pumpkin plants [15]. Analogously, an investigation of the *Wolbachia* infection status of parasitoid wasps showed that the wasps can act as both vectors and hosts for *Wolbachia*, as the parasitoids were found to carry similar *Wolbachia* strains as those found in their hosts [16, 17], and as those found in the other parasitoid species feeding on the same hosts [7]. Although exploring each potential transfer route independently is informative [9, 18, 19], the distribution of *Wolbachia* in the host phylogeny is likely to be the result of a combination of both the host cladogenesis, and diverse horizontal transfer events between host species, the mechanisms of which yet remain to be characterized. Investigating horizontal movements of the endosymbiont between a wider diversity of host species, including species sharing micro or macro-niches, will increase our understanding of the diverse routes used for the HT of *Wolbachia*, and thus broaden our understanding of this symbiont’s global success.

Butterflies in the genera *Mylothris* and *Bicyclus* seem to be an ideal model for studying of the inter-clade transfer of *Wolbachia*. The two genera (belonging to the families Pieridae and Nymphalidae, respectively) have diverged from each other about 97 My ago [20]. They represent two of the most species-rich genera of African butterflies, each including about 100 species [21, 22]. Both *Mylothris* and *Bicyclus* butterflies share similar geographical distributions, covering the Afrotropical region [23–25]. They include specialists for the same types of macro-habitats, from primary forests to forest edges and savannah grasslands [26, 27]. However, despite the syntopic occurrence of many species, the two genera mostly differ in the micro-habitat use. Most distinctively, they inhabit different vertical layers of the habitat [27]. The *Mylothris* species often prefer higher strata [27], where their larval host-plants (mistletoes mostly from Loranthaceae and Santalaceae families) occur [28–30], while the *Bicyclus* species occur predominantly in the undergrowth of the habitat [27], around their grassy larval host-plants (mostly Poaceae family, but sometimes Marantaceae or Zingiberaceae) [31, 32]. The two clades differ also in their adult food resources. Whilst *Mylothris* butterflies are commonly nectaring on various plant species (Tropek, unpublished data), *Bicyclus* are mostly fruit-feeders and sap-suckers and are observed on flowers only occasionally [33, 34]. On the other hand, species from both clades are observed mud-puddling, during which they could interact.

Prior to this study, *Mylothris agathina* was possibly the only *Mylothris* species to be known to carry *Wolbachia* [35, 36]. Earlier, Poulton [37] described an all-female brood in a species he referred to as *M. spica*, in Cameroon. This particular phenotype could be suggestive of an infection with a sex-ratio distorting *Wolbachia* strain, similar to the ones infecting *Acraea encedon*, *A. encedana* [13], or *Hypolimnas bolina* [38], but this has yet to be fully tested. In contrast, a recent study showed that at least 19 *Bicyclus* species carry *Wolbachia* [39]. Many of the strains characterized in the divergent *Bicyclus* species shown high genetic similarity [39], and were also similar to strains described earlier in various insects, including Lepidoptera, from other geographic regions [9, 19, 40]. These patterns are suggestive of the horizontal acquisition of the bacterium between *Bicyclus* species, though the mechanisms of the transfers remained unclear.

We predicted that butterflies belonging to the same genus could share similar strains of *Wolbachia* due to recent common ancestry, and the possibility of HT by the means of hybridization events and shared larval host-plants. We did not expect the same to be true between the two host genera, as the hybridization between individuals of different families is impossible, and as the two genera studied here do not share micro-habitats (as stated above). To further looked at the potential role of geographic distribution and habitat on any particular ecological routes to the transfer of the symbiont between species, we included *Wolbachia* strains previously characterized from any Lepidoptera, any Hymenoptera (many of which could be parasitoid species of Lepidopteran larvae), and any other African arthropods, to the analyses. Finally, we call upon the investigation of more insect communities across the globe, and upon the revision of the current MLST-based *Wolbachia* strain and strain-type (ST) characterization method.

## Results

### *Wolbachia* screening and strain diversity

Out of the 225 *Mylothris* butterflies screened, 70 specimens (31%) were found infected with *Wolbachia*, representing 23 of the 53 species (43%) included in the study. Similarly, 15 out of the 63 *Bicyclus* specimens (24%), representing 10 out of 21 species (47.5%) screened, were infected with *Wolbachia*. This brings the total number of *Bicyclus* species known to carry *Wolbachia* to 23 (19 described by [39], and four new ones in the present study). One of the two *Aphysoneura scapulifascia* specimens included in this study was also found to host *Wolbachia*, while the three *Brakefieldia peitho* specimens were uninfected. We successfully sequenced between three and six *Wolbachia* markers for 66 of the 86 butterflies (77%) found infected with the symbiont.

**Fig. 2:**
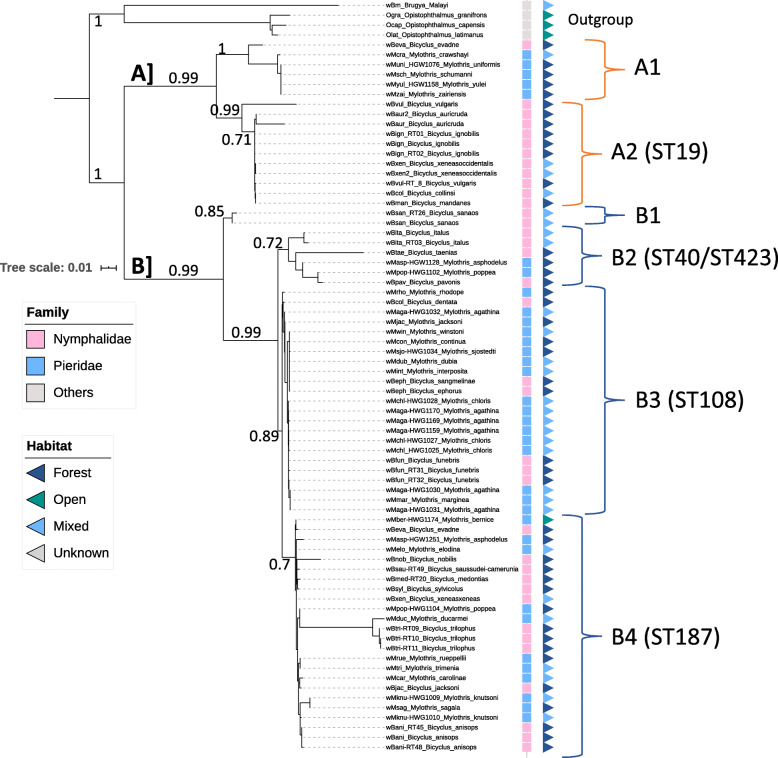
Phylogenetic tree of the *Wolbachia* strains and strain variants characterized from the *Mylothris* and *Bicyclus* butterflies, with habitat type inhabited by the host species, with bootstrap values. The tree was built using the concatenated sequences of the *Wolbachia* MLST and *wsp* markers. Additional *Wolbachia* strains characterized from *Brugya malayi* nematode (D-supergroup strain) and *Opistophthalmus* scorpions (F-supergroup strains) were added as outgroup. Strain variants A1, A2, and B1 to 4, including characterized Strain-Types, are shown on the right side of the figure

There was a higher detectable diversity of B-supergroup than A-supergroup *Wolbachia* strains in the *Mylothris* and also in the *Bicyclus* butterflies (Fig. 2, S1, and S2, and Table S1, Table S2). Most of the infected specimens were infected with B-supergroup *Wolbachia* (N_*Mylothris*_ = 57; N_*Bicyclus*_ = 13; N_*Aphysoneura*_ = 1, or 81.5, 87 and 100% respectively), while the other infected specimens carried A-supergroup *Wolbachia*. Our analyses suggested that the strains clustered within two divergent A-supergroup strains (A1 and A2), and four B-supergroup strains (B1-B4), some belonging to the Strain-Type ST-19, ST-40, ST-108, ST-187, and ST-423 (Fig. 2) [39], and other STs not yet characterized in the pubMLST-*Wolbachia*. However, the *Bicyclus* and *Mylothris* species studied here did not carry any strain of the ST-41, which was previously suggested as highly common in Lepidoptera [9, 40]. The host species *M. uniformis*, *M. yulei*, and *M. asphodelus* were found to carry two infections, each as single infection (i.e. different specimens of the same species carry different *Wolbachia* strains); and we suggest multiple infections in five butterflies (2x *M. agathina* and 3x *M. bernice*), as double peaks in the chromatogrammes from these specimens were observed, even after repeating sequencing on independent PCR products. Finally, the sequencing failed for two *Wolbachia*-infected samples (HWG1_176: *M. crawshayi,* and HWG1_211: *M. asphodelus*; Table S1), which has not allowed us to conclude on the identity of the infection in these specimens*.*

### *Wolbachia* host specificity

From our dataset, there was no effect of cladogenesis on whether two species carry similar *Wolbachia* strains, with no pattern of co-evolution between the butterflies and their respective infections (Fig. 2). For example, The *Wolbachia* strain variants ‘B4’ (Fig. 2) are found in species from at least five divergent Bicyclus species-groups, including the evadne-group, the saussurei-group, the angulosa-group, the trilophus-group and the hewitsoni-group [41]. Additionally, many of the *Wolbachia* strains characterized in the *Mylothris* butterflies (family Pieridae, in blue in Fig. 2) were similar to those from the *Bicyclus* butterflies (family Nymphalidae, in blue in Fig. 2) [39] (Table S3), and to some from other Lepidoptera, or other insects (Figure S3).

Finally, we could not detect any clustering of the strains based on their host habitats (i.e. open savannah versus forest) (Fig. 2, S1, and S2), nor based on their host ecoregions (e.g. Afrotropics versus Oceania) (Figure S3). Strong biases however occur in the dataset used for the present study. There were for example very few *Wolbachia* strains characterized from host species evolving in certain ecoregions available in the PubMLST-*Wolbachia* database, especially from the Neotropics or the Nearctics. Additionally, the dataset is incomplete (i.e. missing data about habitats of the Hymenoptera species).

## Discussion

We predicted that butterflies belonging to the same genus would share similar strains of *Wolbachia* due to recent common ancestry, and the possibility of HT by the means of hybridization events, and/or shared resources. We did not expect the same to be true between genera as any hybridization is impossible, and the butterflies of the two genera considered in this study only share similar macro-habitats (i.e. forest and open savannah), but not micro-habitats (i.e. larval host-plant). Our data did not support co-cladogenesis of *Wolbachia* in the African butterflies, but still partially supported the first prediction. Within each genus, many species carry similar strains to the one found in congeneric species, but not always. However, the same was also true between genera, which contrasts with our second prediction. The occurrence of similar *Wolbachia* strains in both of the two Lepidoptera families (Pieridae and Nymphalidae) is unlikely to occur through shared ancestry, nor through horizontal transfer via the larval host-plants. This is in a clear contrast with the insect communities associated with fleshy mushrooms [14], and pumpkin plants [15]. These results suggest that factors other than the larval host-plants must support the transfer of *Wolbachia* between host species. The study of the horizontal transfer of *Wolbachia* between host species might however be currently skewed by (1) our restricted knowledge of the ecology of each species within insect communities, (2) the strong biases associated with the available *Wolbachia* strain diversity dataset, and (3) the way we characterize the different strains of the bacterium.

As it is the case for many species, especially in the Afrotropics, many aspects of the ecology of the *Mylothris* and *Bicyclus* butterflies remain unfortunately poorly studied. To date, almost all ecological studies of the *Mylothris* butterflies focus on their association at the larval stage to mistletoe plants (e.g. Santalaceae family) [28–30, 42] in their native Afrotropical range [23–25], neglecting other aspects of their life history. There is currently no available comprehensive record, or formal study looking at the community of parasitoid wasps or mite communities associated with any *Mylothris* or *Bicyclus* butterflies. To our knowledge, Gupta et al. [43] provided the only description of *Cotesia pistrinariae* as a parasitoid wasp of *M. chloris*; but it remains unknown whether *C. pistrinariae* could also parasitize any *Bicyclus* species, or vector *Wolbachia* between insect hosts. Our phylogenetic tree suggests several examples of parasitoid wasps sharing similar infection to *Bicyclus* or *Mylothris* butterflies, however in each case the direct contact between the Hymenoptera and the Lepidoptera species are unlikely [9], due to geographical or ecological reasons, or both. For example, despite sharing similar *Wolbachia* strains, the braconid parasitoid wasp *Apanteles chilonis*, an endoparasitoid of the rice stem borer *Chilo suppressalis* [44] in the Palearctic, is unlikely to parasitize *B. vulgaris* or *B. auricruda* in the Afrotropics. Similarly, *Evania appendigaster*, a parasitoid of cockroaches [45], is also unlikely to predate on *B. ignobilis* or *B. xeneas*. Only systematic surveys of the *Wolbachia* strains from species communities, rather than individual species or clades, could potentially offer the material currently lacking for testing how a single strain of *Wolbachia* may occur in highly different hosts and environments. Investigating the *Wolbachia* infection status of the community of endo- and ectoparasites associated with the *Bicyclus* and *Mylothris* butterflies, should thus inform whether these parasites can act as vectors of *Wolbachia* among the two genera of butterflies, as it was previously suggested in other insects, including flies, mosquitoes and ants [7, 19, 46].

*Wolbachia* is known to survive in an extracellular phase in the laboratory for up to a week [47]. Although *Mylothris* and *Bicyclus* larvae use very different host-plants and adult food resources [48, 49], the adult butterflies of both genera have occasionally been observed sucking from the same mud-pools or animal feces. By potential being the only nutrient resources shared by the two genera (Tropek, pers. comm.), mud-pools and feces could thus represent suitable short-term environments supporting the survival of *Wolbachia* until its successful horizontal transfer to a new host niche. This, however, remains to be tested.

Although the origin of *Wolbachia* supergroups A and B is estimated to be 200 My ago (based on whole genome data, [50]), the divergence of the strains within each supergroup is most likely much younger (e.g. estimated around 28 My ago by Ahmed et al. [9] based on the MLST markers only), and does not match the divergence between Pieridae and Nymphalidae butterflies (97 My ago, [20]). This further support our claim that co-cladogenesis is improbable, and strains have not been passed down from their common ancestor or transferred via hybridization events between the butterfly species. Additionally, the ecological links described so far as potential routes for the recent transfer of *Wolbachia* between species can only explain local HTs of the bacterium. Nonetheless, Ahmed et al. [9] found that strain type ST-41, a strain type commonly characterized in butterflies [9, 40], was found in species from Africa (i.e. *Azanus mirza;* Lycaenidae), Japan (i.e. *Eurema hecabe*; Pieridae), Borneo (i.e. *Nacaduba angusta*; Lycaenidae) and North America (i.e. *Celastrina argiolus;* Lycaenidae). Following these results, we show that *Mylothris* and *Bicyclus* butterflies in Africa share similar *Wolbachia* strains to, for example, Lycaenidae from South Africa (with ST-19) or Malaysia (with ST-40) [9], or moths from the Pacific islands [51], and potentially to many other species in between these two geographical regions. None of the geographically distant host species described in these two studies are likely to share the same host-plants, parasitoids nor mite parasites. Despite the lack of a clear understanding of ‘how’, the research community however agrees that the ability of *Wolbachia* to transfer horizontally has without a doubt contributed to the global pandemic of the bacterium [52].

A recent study by Detcharoen et al [53] estimated that, to date, more than 99% of all existing *Wolbachia* strains have yet to be characterized; worse: that strong biases occur in the database. The PubMLST-*Wolbachia* database [18] currently includes over 2000 strains. Out of those, 370 are from Lepidoptera species (18.3%), which is more than for the Coleoptera (92; 4.6%), the Hemiptera (297; 14.7%), and the Hymenoptera (359; 17.8%), but less than the Diptera (473; 23.4%). Thus, strains from particular insect orders, but also host families are more represented. Furthermore, in Lepidoptera for example, most of the *Wolbachia* strains were characterized from species inhabiting the Palearctic ecoregion (*N* = 107; 29%), while very few are from the Afrotropics (*N* = 18; 5%). And this pattern at the ecoregion level is similarly found in the other insect orders, representing another important bias in the PubMLST-*Wolbachia* database. Although the present study brings new data for the Afrotropic region, showing for example that the ST-41 commonly found in Lepidoptera [9], is not found in the *Mylothris* and *Bicyclus*, many biases still remain, and they will continue to impede the comprehensive study of the diversity and geographical distribution of *Wolbachia* strains, as well as our understanding of the mechanisms behind their pandemic.

The commonly applied method to characterized *Wolbachia* strains is based on the sequences of six markers for a maximum length of about 3000 bp [18]. This molecular technique has recently been highly criticized [54]. New studies are pushing towards the use of whole genome data, which seems to more accurately infer *Wolbachia* supergroup phylogeny and origin [50, 55]. Although still rather expensive, whole genome sequencing can not only provide the material to improve our understanding of *Wolbachia* strain diversity, its diversification rates, and its HT, but can also support the investigation of the ecology and evolution of the bacterium, including for example its ability to modify its host phenotype [56], and maybe, one day, its ability to establish in a wide range of host species.

The horizontal transfer of *Wolbachia* between insect hosts was already suggested in the early 90’s [57, 58]. Our study contributes to the growing literature showing that ecological links between species can act as platforms to the between species transfer of the symbiont, however no common understanding of this process and the relative importance of each transfer route has yet been proposed. Furthermore, our study also re-enforces the idea that biases in the dataset, and restrictions in the methodological approaches associated with such study, will, until solved, continue to impede our comprehensive analyses and understanding of the global *Wolbachia* pandemic.

## Methods

### Material

All *Mylothris* specimens used in this study originated from the private collections of Haydon Warren-Gash and Robert Ducarme, and from the African Butterfly Research Institute ‘ABRI’ holding, which were collected under various local collection permits. All *Bicyclus*, *Aphysoneura* and *Brakefieldia* specimens were collected under research permits from the Cameroonian government to Dr. Robert Tropek. Tissue material from 225 adult butterflies from 53 *Mylothris* species [22], 63 specimens from 21 *Bicyclus* species, two specimens of *Aphysoneura scapulifascia*, and three specimens of *Brakefieldia peitho* were included in the present study. The sample size for each species, and country of origin of each specimen can be found in the document available from Zenodo (doi:10.5281/zenodo.3934112).

### Habitats and ecoregions

The world’s terrestrial lands have been divided in eight biogeographic realms, which delineations do not follow countries boundaries, but are defined by the evolutionary history of the organisms they contain [59, 60]. The eight biogeographic realms, here called ecoregions for simplicity, are (1) Afrotropic (Trans-Saharan Africa and Arabia), (2) Antarctic, (3) Australasia (Australia, NewGuinea, and New Zealand), (4) Indo-Malay (Indian subcontinent Southeast Asia and Southern China), (5) Nearctic (North America), (6) Neotropic (South and Central America and the Caribbean), (7) Oceania (South Pacific islands), and (8) Palearctic (Eurasia and North Africa) [60]. Each of these ecoregion covers a wide diversity of biomes, or habitats. The *Mylothris* and *Bicyclus* butterflies evolve only within the Afrotropical region [23–25], but different species are found from either dense primary forests (i.e. forest habitat), forest edges (i.e. mixed habitat), or open savannah grassland habitats (i.e. open habitat) (Fig. 2) [61].

### Molecular work

DNA was extracted from legs from each butterfly following the protocol of a Qiagen DNeasy Blood & Tissue Extraction Kit (Qiagen, USA). We screened all specimens for *Wolbachia*, using *Wolbachia* specific primers amplifying the *wsp* gene (81F/691R, [62]), and three to five of the *Wolbachia* Multi Locus Sequence Typing markers (MLSTs, [18]). All sequences were aligned and manually curated in Geneious R11.0 (http://www.geneious.com, [63]), and submitted to GenBank under the accession codes: MT669957–70007 & MT782897–3039.

### Genetic data from additional *Wolbachia* strains

In order to (I) identify whether the sequences from the *Wolbachia* characterized from our butterfly samples were unique or not to their host species, and (II) characterize any potential route of transfer of the strains between species (Fig. 1), we fished out the sequences of the *wsp* and *MLST* markers, from all Lepidoptera, all Hymenoptera, and all other African arthropods that were available from the PubMLST-*Wolbachia* database by December 2019 [18]. Many of the records from this database were from specimens of the same species and the same population, we thus randomly deleted some of the duplicates to keep a maximum of three of each type. Additionally, we included all *wsp* and MLSTs sequences from *Wolbachia* strains previously characterized from *Bicyclus* species [39] (GenBank IDs: KY658538–52, KY658652, KY658655, and KY658572–90), and those from Malagasy dung beetles [11] (GenBank IDs: MK636654–66), that are not present in the PubMLST-*Wolbachia* database. The full list of specimens and sequences included in this study can be retrieved from Zenodo (doi: 10.5281/zenodo.3934112).

### Phylogenetic analyses

The sequences of the six *Wolbachia* markers were concatenated in the following order: *coxA, fbpA, ftsZ, gatB, hcpA,* and *wsp*, for a maximum alignment of 3149 bp. Each tree was built in CIPRES [64] using RAxML-XSEDE [65] with the Gamma+I parameter. Tree visualization and figures were done with FigTree (http://tree.bio.ed.ac.uk/software/figtree/) and ITOL [66, 67] using the bipartitions output trees produced by RAxML.

## Supplementary information

**Additional file 1: Table S1**: Divergence rate (%) of the *wsp* marker between the 11 *Wolbachia* strains and strain variants characterized from *Mylothris* butterfly species. A-supergroup *Wolbachia* strains are shown in pink, B-*Wolbachia* in blue**.** Inside cell colors vary in accordance with degree of similarity (white: less than 75% similarity, gray: between 75 & 97% similarity, dark-gray: more than 97% similarity). **Table S2**: Divergence rate (%) of the *wsp* marker between the 14 *Wolbachia* strains and strain variants characterized from *Bicyclus* butterfly species (as characterized in this study and by (Duplouy and Brattstrom [39])). A-supergroup strains are shown in pink, B-*Wolbachia* in blue. All variants share the same color**.** Inside cell colors vary and in accordance with degree of similarity (white: less than 75% similarity, gray: between 75 & 97% similarity, dark-gray: more than 97% similarity). **Table S3**: Divergence rate (%) between the *Wolbachia* strains and strain variants characterized from the *Mylothris* butterflies and the *Bicyclus* butterflies (as characterized in this study and by (Duplouy and Brattstrom [39])). Central cells colored in accordance with degree of similarity between strains (white: less than 75% similarity, gray: between 75 and 97% similarity, dark-gray: more than 97% similarity). **Figure S1**: Rooted phylogenetic relationships of the concatenated MLST and *wsp* genes sequences from the different *Wolbachia* characterized from the *Mylothris* butterflies, with bootstrap values. Additional *Wolbachia* strains characterized from *Brugya malayi* (D-supergroup strain) and from *Opistophthalmus* scorpions (F-supergroup strains) were added as outgroup. Habitat of the host is shown in right-circle. **Figure S2**: Rooted phylogenetic relationships of the concatenated MLST and *wsp* genes sequences from the *Bicyclus* butterflies, with bootstrap values. Additional *Wolbachia* strains characterized from *Brugya malayi* (D-supergroup strain) and from *Opistophthalmus* scorpions (F-supergroup strains) were added as outgroup. Habitat of the host is shown in right-circle. **Figure S3**: Phylogenetic tree of all available *Wolbachia* strains and strain variants characterized from Lepidoptera, Hymenoptera, and all other African arthropods. The tree was built using the concatenated sequences of the *Wolbachia* MLST and *wsp* markers. Colored squares, circles and triangles on the right provide the family, ecoregion and habitat of the hosts, respectively. Dataset includes strains described in the present study, as well as strains from *Bicyclus* butterflies as in [39], from Malagasy Nanos dung-beetles as in [11], and all pubMLST-registered strains from Lepidoptera, Hymenoptera and African arthropods [18]. *Wolbachia* strains characterized from *Brugya malayi* nematode (D-supergroup strain) and from *Opistophthalmus* scorpions (F-supergroup strains) were used as outgroup.

## Data Availability

The dataset supporting the conclusions of this article is available in the Zenodo repository, https://zenodo.org/record/3934112#.X1YrqlBS_BI.

## Abbreviation

ST: Strain Type
