# Supplementary material for: Towards unravelling Wolbachia global exchange: a contribution from the Bicyclus and Mylothris butterflies in the Afrotropics
Source: BMC Microbiol. 2020 Oct 20;20:319. doi: 10.1186/s12866-020-02011-2 (PMC7576836; doi:10.1186/s12866-020-02011-2)
Supplement: Supplementary file 1 — Additional file 1: Table S1: Divergence rate (%) of the wsp marker between the 11 Wolbachia strains and strain variants characterized from Mylothris butterfly species. A-supergroup Wolbachia strains are shown in pink, B-Wolbachia in blue. Inside cell colors vary in accordance with degree of similarity (white: less than 75% similarity, gray: between 75 & 97% similarity, dark-gray: more than 97% similarity). Table S2: Divergence rate (%) of the wsp marker between the 14 Wolbachia strains and strain variants characterized from Bicyclus butterfly species (as characterized in this study and by (Duplouy and Brattstrom [39])). A-supergroup strains are shown in pink, B-Wolbachia in blue. All variants share the same color. Inside cell colors vary and in accordance with degree of similarity (white: less than 75% similarity, gray: between 75 & 97% similarity, dark-gray: more than 97% similarity). Table S3: Divergence rate (%) between the Wolbachia strains and strain variants characterized from the Mylothris butterflies and the Bicyclus butterflies (as characterized in this study and by (Duplouy and Brattstrom [39])). Central cells colored in accordance with degree of similarity between strains (white: less than 75% similarity, gray: between 75 and 97% similarity, dark-gray: more than 97% similarity). Figure S1: Rooted phylogenetic relationships of the concatenated MLST and wsp genes sequences from the different Wolbachia characterized from the Mylothris butterflies, with bootstrap values. Additional Wolbachia strains characterized from Brugya malayi (D-supergroup strain) and from Opistophthalmus scorpions (F-supergroup strains) were added as outgroup. Habitat of the host is shown in right-circle. Figure S2: Rooted phylogenetic relationships of the concatenated MLST and wsp genes sequences from the Bicyclus butterflies, with bootstrap values. Additional Wolbachia strains characterized from Brugya malayi (D-supergroup strain) and from Opistophthalmus scorpions (F-supergroup stra [file 12866_2020_2011_MOESM1_ESM.zip › SuppMaterials-Mylothris-Aug2020.docx]

**Supplementary materials**

**Table S1: Divergence rate (%) of the *wsp* marker between the 11 *Wolbachia* strains and strain variants characterized from *Mylothris* butterfly species.** A-supergroup *Wolbachia* strains are shown in pink, B-*Wolbachia* in blue**.** Inside cell colors vary in accordance with degree of similarity (white: less than 75% similarity, gray: between 75 & 97% similarity, dark-gray: more than 97% similarity).

| **Host species (N=)** | **id** | **Divergence (%)** | | | | | | | | | |
| --- | --- | --- | --- | --- | --- | --- | --- | --- | --- | --- | --- |
|  |  | **2** | **3** | **4** | **5** | **6** | **7** | **8** | **9** | **10** | **11** |
| *M. alluaudi* (2)  *M. uniformis* (5)  *M. yulei* (1) | **1** *w*Mall_A  *w*Muni_A  *w*Myul_A | 99,1 | 75,3 | 74,4 | 74,4 | 74,5 | 75,1 | 74,8 | 74,6 | 74,6 | 72,4 |
| *M. crawshayi* (1) | **2:** *w*Mcra_A | - | 75,4 | 74,6 | 74,6 | 74,7 | 75,3 | 75,0 | 74,8 | 74,8 | 72,6 |
| *M. agathina* (5)  *M. chloris* (4)  *M. dubia* (4)  *M. interposita* (1)  *M. marginea* (2)  *M. sjostedti* (3)  *M. winstoni* (1)  *M. yulei* (1) | **3:** *w*Maga_B  *w*Mchl_B  *w*Mdub_B  *w*Mint_B  *w*Mmar_B  *w*Msjo_B  *w*Mwin_B  *w*Myul2_B |  | - | 98,4 | 99,2 | 99,0 | 98,9 | 98,4 | 98,8 | 99,6 | 80,4 |
| *M. poppea* (2) | **4:** *w*Mpop_B |  |  | - | 99,2 | 99,4 | 99,2 | 98,6 | 99,2 | 98,0 | 80,8 |
| *M. sagala* (4)  *M. knutsoni* (2) | **5:** *w*Msag_B  *w*Mknu_B |  |  |  | - | 99,8 | 99,6 | 99,2 | 99,6 | 98,8 | 80,0 |
| *M. asphodelus* (5)  *M. bernice* (4)  *M. elodina* (2) | **6:** *w*Masp_B  *w*Mber_B  *w*Melo_B |  |  |  |  | - | 99,8 | 99,4 | 99,8 | 99,6 | 80,2 |
| *M. trimenia* (3)  *M. rueppellii* (1) | **7:** *w*Mtri_B  *w*Mrue_B |  |  |  |  |  | - | 99,2 | 99,6 | 98,4 | 80,0 |
| *M. carolinae* (1) | **8:** *w*Mcar_B |  |  |  |  |  |  | - | 99,2 | 98,0 | 79,6 |
| *M. rhodope* (1) | **9:** *w*M*rho_B* |  |  |  |  |  |  |  | - | 98,4 | 80,5 |
| *M. agathina* (2)  *M. marginea* (1) | **10:** *w*Maga_B  *w*Mmar_B |  |  |  |  |  |  |  |  | - | 80,4 |
| *M. ducarmei* (2) | **11:** *w*Mduc_B |  |  |  |  |  |  |  |  |  | - |

**Table S2: Divergence rate (%) of the *wsp* marker between the 14 *Wolbachia* strains and strain variants characterized from *Bicyclus* butterfly species (as characterized in this study and by (Duplouy and Brattstrom 2018)).** A-supergroup strains are shown in pink, B-*Wolbachia* in blue. All variants share the same color**.** Inside cell colors vary and in accordance with degree of similarity (white: less than 75% similarity, gray: between 75 & 97% similarity, dark-gray: more than 97% similarity).

| **Infected species (N)** | **id** | **Divergence (%)** | | | | | | | | | | | | |
| --- | --- | --- | --- | --- | --- | --- | --- | --- | --- | --- | --- | --- | --- | --- |
|  |  | **13** | **14** | **15** | **3** | **4** | **6** | **16** | **17** | **18** | **19** | **29** | **21** | **22** |
| *B. mandanes* (1)  *B. collinsi* (1) | **12:** *w*Bman_A  *w*Bcol_A | 99,7 | 99,8 | 78,0 | 72,8 | 72,8 | 72,8 | 72,7 | 72,8 | 72,6 | 72,5 | 72,8 | 74,4 | 72,0 |
| *B. auricruda* (2) | **13:** *w*Baur_A  *w*Baur2_A | - | 99,8 | 78,0 | 72,9 | 72,8 | 72,9 | 72,7 | 72,8 | 72,7 | 72,6 | 72,9 | 74,7 | 71,8 |
| *B. xeneas occidentalis* (2)  *B.ignobilis* (3) | **14:** *w*Bxen_A  *w*Bign_A  *w*Bxen2_A |  | - | 78,2 | 73,1 | 72,9 | 73,1 | 72,8 | 73,0 | 72,9 | 72,8 | 73,1 | 74,7 | 71,8 |
| B. evadne (1) | **15:** *w*Beva_A |  |  | - | 77,0 | 77,2 | 77,4 | 77,2 | 77,5 | 77,2 | 77,2 | 77,4 | 76,7 | 73,8 |
| *B. sangmelinae*  *B. funebris*  *B. ephorus* | **3:** *w*Bsang_B  *w*Beph_B  *w*Bfun_B |  |  |  | - | 98,3 | 99,1 | 99,1 | 98,9 | 98,8 | 98,9 | 99,1 | 79,1 | 81,8 |
| *B. pavonis* | **4:** *w*Bpav_B |  |  |  |  | - | 99,2 | 99,1 | 99,3 | 98,9 | 99,0 | 98,8 | 77,9 | 82,1 |
| *B. xeneas xeneas*  *B. saussudei*  *B. nobilis*  *B. medontas* | **6:** *w*Bxen_B  *w*Bsau_B  *w*Bnob_B  *w*Bmed_B |  |  |  |  |  | - | 99,6 | 99,8 | 99,6 | 99,8 | 99,6 | 78,8 | 80,8 |
| *B. sylvicolus* | **16:** *w*Bsyl_B |  |  |  |  |  |  | - | 99,5 | 99,3 | 99,5 | 99,3 | 78,6 | 80,8 |
| *B. jacksoni* | **17:** *w*Bjac_B |  |  |  |  |  |  |  | - | 99,5 | 99,6 | 99,5 | 78,8 | 81,0 |
| *B. evadne* | **18:** *w*Beva_B |  |  |  |  |  |  |  |  | - | 99,5 | 99,3 | 78,6 | 81,2 |
| *B. dentata* | **19:** *w*Bden_B |  |  |  |  |  |  |  |  |  | - | 99,5 | 78,5 | 80,6 |
| *B. anisops* | **20:** *w*Bani_B |  |  |  |  |  |  |  |  |  |  | - | 78,4 | 80,4 |
| *B. trilophus* | **21:** *w*Btri_B |  |  |  |  |  |  |  |  |  |  |  | - | 83,3 |
| *B. taenias* | **22:** *w*Btae_B |  |  |  |  |  |  |  |  |  |  |  |  | - |

**Table S3: Divergence rate (%) between the *Wolbachia* strains and strain variants characterized from the *Mylothris* butterflies and the *Bicyclus* butterflies (as characterized in this study and by (Duplouy and Brattstrom 2018))**. Central cells colored in accordance with degree of similarity between strains (white: less than 75% similarity, gray: between 75 and 97% similarity, dark-gray: more than 97% similarity).

| **Strain** | **1:** *w*Muni_A | **2:** *w*Mcra_A | **5:** *w*Msag_B | **7:** *w*Mtri_B | **8:** *w*Mcar_B | **9:** *w*Mrho*_B* | **10:** *w*Maga_B | **11:** *w*Mduc_B |
| --- | --- | --- | --- | --- | --- | --- | --- | --- |
| **12:** *w*Bman_A | 73,1 | 73,5 | 70,2 | 70,2 | 70,4 | 70,3 | 70,5 | 70,8 |
| **13:** *w*Baur_A | 73,5 | 73,9 | 70,2 | 70,2 | 70,4 | 70,3 | 70,5 | 71,0 |
| **14:** *w*Bxen_A | 73,3 | 73,7 | 70,4 | 70,4 | 70,5 | 70,5 | 70,7 | 71,0 |
| **15:** *w*Beva_A | 86,3 | 86,7 | 76,7 | 76,7 | 76,7 | 77 | 76,6 | 74,1 |
| **3:** *w*Bsang_B | 75,6 | 75,8 | 99,2 | 98,9 | 98,4 | 98,8 | 99,6 | 80,9 |
| **4:** *w*Bpav_B | 75,4 | 75,6 | 99,2 | 99,2 | 98,8 | 99,2 | 98 | 81,3 |
| **6:** *w*Bxen_B | 74,8 | 75 | 99,8 | 99,8 | 99,4 | 99,8 | 98,6 | 80,5 |
| **16:** *w*Bsyl_B | 74,5 | 74,7 | 99,4 | 99,4 | 99 | 99,4 | 98,6 | 80,4 |
| **17:** *w*Bjac_B | 75 | 75,1 | 99,6 | 99,6 | 99,2 | 99,6 | 98,4 | 80,7 |
| **18:** *w*Beva_B | 75,4 | 75,6 | 99,4 | 99,4 | 99,4 | 99,4 | 98,2 | 80,3 |
| **19:** *w*Bden_B | 74,5 | 74,7 | 99,6 | 99,6 | 99,2 | 99,6 | 98,4 | 80,2 |
| **20:** *w*Bani_B | 74,8 | 75 | 99,8 | 99,4 | 99 | 99,4 | 98,6 | 80,1 |
| **21:** *w*Btri_B | 72,9 | 73,2 | 80,6 | 81,2 | 80,2 | 80,8 | 80,8 | 99,4 |
| **22:** *w*Btae_B | 73,5 | 73,7 | 75,9 | 75,9 | 75,7 | 75,9 | 76,7 | 82,0 |

**Figure S1:** **Rooted phylogenetic relationships of the concatenated MLST and *wsp* genes sequences from the different *Wolbachia* characterized from the *Mylothris* butterflies,** with bootstrap values. Additional *Wolbachia* strains characterized from *Brugya malayi* (D-supergroup strain) and from *Opistophthalmus* scorpions (F-supergroup strains) were added as outgroup. Habitat of the host is shown in right-circle.

**Figure S2:** **Rooted phylogenetic relationships of the concatenated MLST and *wsp* genes sequences from the *Bicyclus* butterflies,** with bootstrap values. Additional *Wolbachia* strains characterized from *Brugya malayi* (D-supergroup strain) and from *Opistophthalmus* scorpions (F-supergroup strains) were added as outgroup. Habitat of the host is shown in right-circle.

**Figure S3: Phylogenetic tree of all available *Wolbachia* strains and strain variants characterized from Lepidoptera, Hymenoptera, and all other African arthropods**. The tree was built using the concatenated sequences of the *Wolbachia* MLST and *wsp* markers. Colored squares, circles and triangles on the right provide the family, ecoregion and habitat of the hosts, respectively. Dataset includes strains described in the present study, as well as strains from *Bicyclus* butterflies as in [39], from Malagasy Nanos dung-beetles as in [11], and all pubMLST-registered strains from Lepidoptera, Hymenoptera and African arthropods [18]. *Wolbachia* strains characterized from *Brugya malayi* nematode (D-supergroup strain) and from *Opistophthalmus* scorpions (F-supergroup strains) were used as outgroup.
